# Full-Length 16S rRNA Gene Amplicon and Metagenome Taxonomic Profiling of Beneficial Microbes in Poultry and Swine Probiotic Product

**DOI:** 10.1128/mra.00690-22

**Published:** 2022-09-26

**Authors:** Adison Khongchatee, Thidathip Wongsurawat, Kantgamon Suriye, Piroon Jenjaroenpun, Pimlapas Leekitcharoenphon, Worarat Kruasuwan, Suporn Foongladda

**Affiliations:** a Division of Bioinformatics and Data Management for Research, Research Group and Research Network Division, Research Department, Faculty of Medicine Siriraj Hospital, Mahidol University, Bangkok, Thailand; b Siriraj Long-read Lab (Si-LoL), Faculty of Medicine Siriraj Hospital, Bangkok, Thailand; c Department of Microbiology, Faculty of Medicine Siriraj Hospital, Mahidol University, Bangkok, Thailand; d Research Group for Genomic Epidemiology, National Food Institute, Technical University of Denmark, Kongens Lyngby, Denmark; Indiana University, Bloomington

## Abstract

Analysis of feed supplements can highlight microbial diversity and the prevalence of antimicrobial resistance (AMR), allowing users to monitor the safety of their animals. The 16S amplicon and metagenomic data generated by nanopore sequencing revealed that *Bacillus* was the dominant prokaryote, and AMR genes were detected in the animal probiotic products.

## ANNOUNCEMENT

Antibiotics and pharmaceutical compounds were widely utilized to alter the intestinal microbiome and increase production and animal development (1). However, the unregulated usage of those chemicals probably resulted in drug resistance, endangering both animal and human health (2). Even though many probiotics are recognized as safe and used as animal growth promoters (3, 4), little research has been done on microbial diversity and the spread of antimicrobial resistance (AMR). In this study, microbial diversity and AMR profiling were evaluated from 16S amplicon and shotgun data of animal probiotic product generated by Oxford Nanopore Technologies (ONT).

Animal probiotic products were purchased from a Thais market in January 2022 and kept at room temperature before further analysis. Genomic DNA (gDNA) was extracted from 10 g of the product by resuspension with 20 mL of phosphate-buffered saline (PBS). The upper phase was centrifuged for 15 min at 4,500 rpm and the pellet was used for gDNA extraction using the ZymoBIOMICS DNA miniprep kit protocol (D4300; Zymo Research, USA). For the metagenome, 150 ng of gDNA was used for library preparation using a rapid barcoding kit (RBK004; ONT, UK) by cleavage with transposase enzyme and finally ligated with an adapter. For the 16S amplicon, 20 ng of gDNA was amplified using 27F and 1492R primers with LongAmp *Taq* 2× master mix (New England Biolabs, UK) under the following conditions: 95°C for 1 min and 25 cycles of 95°C for 20 s, 55°C for 30 s, and 65°C for 2 min, followed by 65°C for 5 min. The library was prepared using the 16S barcoding kit protocol (SQK-RAB204; ONT, UK).

Next, the library was loaded into an R9.4.1 flow cell (FLO-MIN106) and sequenced using MinION (Mk1C) with the default setting. Guppy v6.0.1 with the superaccuracy mode was used for base calling and quality control studies (5). Porechop v0.2.4 (https://github.com/rrwick/Porechop) was applied to remove adapters and barcodes. NanoPlot v1.20.0 was used to evaluate read quality (5). Read quality scores of >10 with at least a 1,000-bp read length for the 16S amplicon and >9 with at least a 200-bp read length for shotgun sequencing were kept using NanoFilt v2.8.0 (5) for taxonomic classification and AMR detection by One Codex, a web-based data platform for microbial taxonomic classification and functional prediction (6).

**FIG 1:**
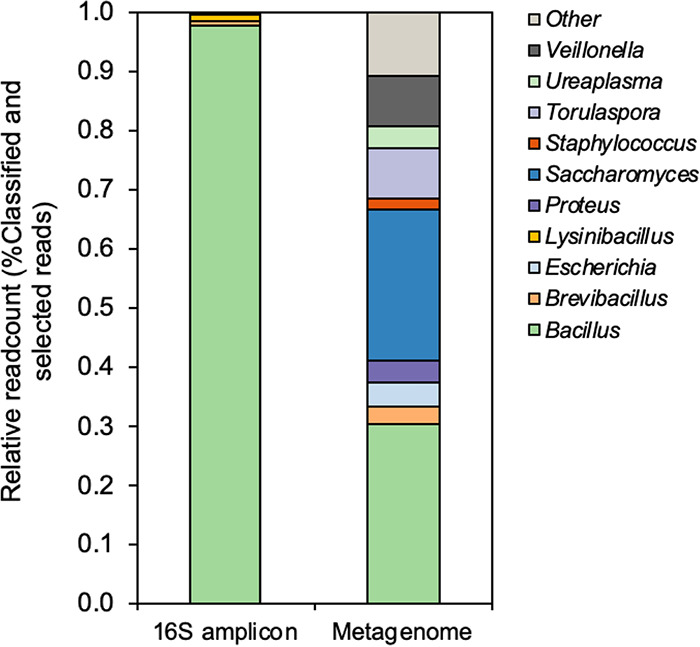
Taxonomic profiling of poultry and swine probiotic product at the genus level from the 16S amplicon and metagenome. Each bar represents the relative frequency of each microbial genus.

The 16S amplicon yielded 173,824 reads with 1,455-bp mean read length, whereas the metagenome generated 322,464 reads and a read length *N*_50_ value of 3,435 bp (Table 1). High-quality reads were taxonomically identified against the NCBI RefSeq Targeted Loci database covering rRNA and internal transcribed spacer (ITS) genes (6). Our results revealed that *Bacillus* and *Brevibacillus* were the most abundant genera in both 16S amplicon and metagenomic data. *Lysinibacillus* was found in only 16S amplicon data, whereas Escherichia, Proteus, *Saccharomyces*, Staphylococcus, *Torulaspora*, *Ureaplasma*, and *Veillonella* were particularly found in shotgun data (Fig. 1). For AMR gene analysis, the sequencing reads were mapped to the reference marker sequences for 478 AMR genes across 28 antibiotic classes (6). Two AMR genes, *aad*(K) (94.7% identity) and *erm*(D) (93% identity), were detected in the metagenomic data.

**TABLE 1:** 16S amplicon and metagenome statistics of poultry and swine probiotic product

Sequence type	Total no. of reads	No. of quality bases (bp)	*N*_50_ (bp)	Mean read length (bp)	SRA accession no.
16S amplicon	173,824	252,925,198	1,464	1,455	SRR18682825
Metagenome	322,464	468,747,357	3,435	1,453	SRR18682823

### Data availability.

The raw sequencing data are available at the NCBI Sequence Read Archive (SRA) under BioProject PRJNA823500 with accession numbers SRR18682825 (16S amplicon) and SRR18682823 (metagenomic sequencing).
